# Seroprevalence of anti-HDV Ab and socioepidemiological characteristics among HBsAg-positive blood donors in Charmahal-o-Bakhtiyari province, Iran: Anti-HDV among HBsAg-positive blood donors

**Published:** 2011-02-01

**Authors:** Elahe Tajbakhsh, Behnam Abbasian, Abolghasem Esmaeili, Mohaddeseh Behjati

**Affiliations:** 1 Department of Microbiology, Faculty of Veterinary Medicine, Islamic Azad University of Shahrekord, Shahrekord, IR Iran; 2 Molecular and Developmental Division, Department of Biology, Faculty of Science, University of Isfahan, Isfahan, IR Iran; 3 Department of Internal Medicine, Isfahan University of Medical Sciences, Isfahan, IR Iran

**Keywords:** Hepatitis D virus, Blood donor, Iran, Seroprevalence

Dear Editor,

Several lines of evidence imply that hepatitis D virus (HDV) infection has a worldwide distribution. It is quite common in some areas such as Russia, Romania, Southern Italy, Mediterranean countries, Africa, South American (Venezuela, Columbia, Brazil, and Ecuador) and China [1]. We conducted this study to determine the seroprevalence of HDV in Shahrekord, a small city in Charmahal-o-Bakhtiyari province, West of Iran and compared it with the results of studies carried out in the larger cities in Iran. We studied 11,472 (9602 men and 1870 women) volunteer blood donors. All participants completed a questionnaire including sociodemographic data, past medical history of symptomatic hepatitis, gender, age, educational level and their residence (urban vs. rural). Blood samples taken from donors were checked for HBsAg and IgM against HBcAgTable 1. In HBsAg-positive cases, anti-HDV antibody level was determined at the baseline using frozen serum specimens by enzyme-linked immunosorbent assay (ELISA). The results were compared between HDV-positive and -negative cases. This study was approved by the Research Ethics Committee of Shahrekord Azad University and conducted with help of Blood Transfusion Organization. Data were analyzed with SPSS ver 9.0 (SPSS Inc., Chicago, IL) using x(2) test. Continuous variables are presented as Mean±SD.The studied participants had a mean age of 27 (range: 18-64) years. There were 8849 (83.7%) men and 8151 of them (77.1%) were married. The rate of tattooing, blood letting and suspicious sexual contacts were 34%, 17% and 3%, respectively. HBsAg was found positive in 0.78% of participants (90 of 11,472-67 men and 13 women); 13 (0.11%) and 6 (0.05%) of whom were seropositive for HBc-Ab and HDV-Ab, respectively. Among seropositive samples for HBsAg, 6.6% were seropositive for anti-HDV IgG. No significant association was observed between HDV seropositivity and sociodemographic characteristics.

**Table 1:** Serological data of the participant blood donors

**Indicator**	**Positive cases**	**Percentage of total**	**Percentage of HBS-Ag + cases**
**HBsAg a**	90	0.78	100%
**IgM HBc b**	13^d^	0.11	14.4%
**Anti-HDV c**	6 d	0.05	6.6%

^a^ Hepatitis B surface antigen (HBSAg)

^b^ Hepatitis B core antigen (IgM-HBc)

^c^ Hepatitis D virus (HDV)

^d^ 13 and 6 were positive from 90 HBsAg-positive cases

In Iran, seroprevalence of HDV infection varies from 2.4% in blood donors to 10% in chronic liver disease patients [2]. In asymptomatic carriers of HBsAg from Jordan, Kuwait, Saudi Arabia, and Turkey the seroprevalence rate was reported as 2%, 31%, 3.3%, 5.2%, respectively [3]. Its prevalence in acute hepatitis patients from Egypt, Jordan, Kuwait and Tajikestan was reported to be 16.94%, 16%, 4% and 9.2%, respectively. The rate in chronic liver disease patients from Yemen, Turkey, Jordan and Egypt was 2%, 32.7%, 23% and 23.53%, respectively [3]. The seroprevalence rate of 6.6% for anti-HDV IgG among volunteer blood donors referred to Shahrekord Blood Bank is much less than that reported earlier.
